# Developing an Emotion- and Memory-Processing Group Intervention for PTSD with complex features: a group case series with survivors of repeated interpersonal trauma

**DOI:** 10.1080/20008198.2018.1495980

**Published:** 2018-07-30

**Authors:** Georgina Clifford, Richard Meiser-Stedman, Rebecca D. Johnson, Caitlin Hitchcock, Tim Dalgleish

**Affiliations:** a Medical Research Council Cognition and Brain Sciences Unit, Cambridge, UK; b Department of Clinical Psychology, University of East Anglia, Norwich Research Park, Norwich, Norfolk, UK; c The Haven Sexual Assault Referral Centre, St. Mary’s Hospital, Paddington, London, UK; d Complex Care Team, Halliwick Centre, St Ann’s Hospital, London, UK; e Cambridgeshire and Peterborough NHS Foundation Trust, Cambridge, UK

**Keywords:** Posttraumatic stress disorder, PTSD, complex PTSD, CPTSD, emotion regulation, trauma-focused therapy, group therapy, group case series, Trastorno de Estrés Postraumático, TEPT, TEPT Complejo, TEPTC, Regulación emocional, Terapia Focalizada en el Trauma, Terapia Grupal, Series de casos grupales, 创伤后应激障碍, PTSD, 复杂PTSD, CPTSD, 情绪调节, 聚焦创伤疗法, 团体治疗, 团体案例系列, • Reports a small (*N =* 15) case series of an Emotion- and Memory-Processing Group Intervention.• Primary aim was to establish acceptability and feasibility; secondary aim was to explore treatment efficacy.• Treatment achieved a 76% completion rate with one drop-out.• Large effect sizes demonstrated for PTSD severity (*d* = 1.18) and severity of complex features (*d* = 0.96).

## Abstract

Individuals who experience repeated interpersonal trauma exposure often present with posttraumatic stress disorder (PTSD) with more complex features. There is currently no consensus regarding whether current evidence-based interventions for PTSD need to be tailored to better account for these complex features. However, one recommended adaptation is to adopt a phase-based or sequenced approach involving three phases, each with a distinct function. This paper describes the development of a 12-session Emotion- and Memory-Processing Group Programme, adapted from Cloitre’s Skills Training in Affective and Interpersonal Regulation (STAIR) phase-based treatment protocol. A single case series provided a preliminary examination of the group-based intervention’s efficacy for three groups of women with a history of repeated interpersonal trauma and PTSD with complex features (*N *= 15; age 19–46 years) at The Haven Sexual Assault Referral Centre in London. Results revealed significant reductions in: PTSD, complex features of PTSD, and depression, along with improvements in process measures of maladaptive cognitions and emotion processing. Results from this case series demonstrate that an Emotion- and Memory-Processing Group Programme holds promise for treating individuals with a history of interpersonal trauma in outpatient settings, and provides evidence to warrant the completion of a feasibility trial.

## Abstract (es)

Las personas que experimentan una exposición al trauma interpersonal de manera repetida a menudo presentan un Trastorno de Estrés Postraumático (TEPT) con características más complejas. Actualmente, no hay consenso respecto a la necesidad de adaptar las intervenciones para el TEPT basadas en la evidencia disponible, con el fin de considerar mejor estas características complejas. Sin embargo, una adaptación recomendada es adoptar un abordaje basado en fases, o secuenciado, que involucra tres fases, cada una con una función distinta. Este artículo describe el desarrollo de un Programa Grupal de Procesamiento de Emociones y Memoria de 12 sesiones, adaptado del protocolo de tratamiento basado en fases del Entrenamiento de Habilidades en Regulación Afectiva e Interpersonal (STAIR) de Cloitre. Una serie de casos únicos proporcionó un examen preliminar de la eficacia de la intervención basada en grupos para tres grupos de mujeres con una historia de trauma interpersonal repetido y TEPT con características complejas (*N =* 15, edades 19 años-46 años) en el Centro de Derivación de Agresión Sexual de Haven en Londres. Los resultados revelaron reducciones significativas en: TEPT, características complejas del TEPT, y depresión, junto con mejoras en medidas de procesos de cogniciones desadaptativas y procesamiento de emociones. Los resultados de esta serie de casos demuestran que el Programa de Grupo de Procesamiento en Emoción y Memoria es prometedor para tratar individuos con una historia de trauma interpersonal en contextos ambulatorios, y proporciona evidencia para garantizar la realización de un ensayo de viabilidad.

## Abstract (zh)

反复经历人际创伤暴露的个体经常出现具有复杂特征的创伤后应激障碍（PTSD）。目前还没有一致的结论确认是否需要针对这些复杂特征修订PTSD的循证干预措施。尽管如此，受到建议采用的调整方法是使用阶段式或循序干预的方法，其中包括三个功能各异的阶段。本文描述了一个12期的情绪和记忆加工团体干预项目的开发，该干预项目是根据Cloitre的情感和人际调节技能阶培训（STAIR）的阶段性治疗大纲改编。在伦敦的 Haven 性侵转诊中心（Haven Sexual Assault Referral Centre）招募了三组有反复人际创伤和复杂 PTSD 的女性（*N =* 15，年龄19岁-46岁），作为一个案例系列提供了对该团体干预有效性的初步检验。结果显示了PTSD、复杂PTSD和抑郁的显著减轻，伴随着适应不良的认知和情绪加工的改进。本案例系列的结果表明，情绪和记忆加工团体干预项目有望在门诊治疗具有人际创伤史的个体，同时还提供了证据保证可行性试验的完成。

## 1. Introduction

Individuals presenting with posttraumatic stress disorder (PTSD) are not a homogenous group. Those who experience repeated interpersonal trauma, such as sexual and domestic violence, and abuse in childhood often present with PTSD with more complex features (Karatzias et al., 2017; Powers et al., 2017) than individuals exposed to single-incident traumas (Herman, 1997). Proposed diagnostic criteria for Complex PTSD (CPTSD) in the ICD-11 (due to be published in 2018) include the defining criteria of PTSD (re-experiencing, avoidance, numbing, and hyperarousal), in addition to the presence of at least one symptom in each of three self-organization features: affect dysregulation, negative self-concept, and interpersonal disturbance. The affective domain problems are characterized by emotion dysregulation, including alterations in attention and consciousness (e.g. dissociation, depersonalization, and derealization). Negative self-concept criteria include persistent beliefs about oneself as diminished, defeated, or worthless, and interpersonal disturbances are defined by persistent difficulties in sustaining relationships (Briere, Kaltman, & Green, 2008; Cloitre, Garvert, Brewin, Bryant, & Maercker, 2013; Cloitre et al., 2009).

There is contention in the literature regarding whether PTSD and CPTSD can be conceptualized as different disorders (see Resick et al., 2012, for discussion), and there is currently no consensus regarding whether tailoring current evidence-based interventions for PTSD (e.g. eye movement desensitization and reprocessing [EMDR], trauma-focussed cognitive behavioural therapy [CBT]) for complex features will improve treatment outcomes (Cloitre et al., 2012; Van Minnen, Harned, Zoellner, & Mills, 2012). A number of authors propose that trauma-focused treatments can be offered to those who have experienced repeated interpersonal trauma without any major modifications (e.g. Cook, Schnurr, & Foa, 2004; Resick, Nishith, & Griffin, 2003; Van Minnen et al., 2012). Others propose that outcomes for complex presentations can be improved using a phase-based or sequenced approach involving three phases, each with a distinct function (e.g. Cloitre et al., 2012). Phase one focuses on ensuring the individual’s safety, reducing symptoms, and increasing important emotional, social, and psychological competencies. Phase two focuses on processing the unresolved aspects of the individual’s memories of traumatic experiences. Phase three involves consolidation of treatment gains to facilitate engagement in relationships, work or education, and community life. At present, there is no clear evidence-base to demonstrate consistently superior treatment effects for the use of a standard or phase-based approach to treating complex features (e.g. Wagenmans, Van Minnen, Sleijpen, & De Jongh, 2018; Bongaerts, Van Minnen, & De Jongh, 2017; Van Minnen et al., 2012).

Other elements of treatment format are also in need of further examination, including the use of group-based delivery. There are a number of advantages to offering group-based treatment, including a shared focus on resolution of symptoms through psychoeducation and skills training, which can be effective in terms of both time and cost. Relative to individual therapy, group interventions may be particularly useful for survivors of repeated interpersonal trauma, to normalize symptoms, foster social support, and enable observational learning (Dorrepaal et al., 2012; Zlotnick et al., 1997). Group therapy can provide an opportunity for individuals to experience, explore, and work through individual difficulties with others perceived to be in some way similar to oneself (e.g. Foy et al., 2000), and help them to make sense of their own experiences and responses to trauma (Klein & Schermer, 2000). In turn, this can reduce self-blame and feelings of disconnection or isolation from others (e.g. Johnson & Lubin, 2000).

Group therapy for PTSD is not currently included in any treatment guidelines (e.g. Forbes et al., 2010). However, the group-based format is commonly used in health care settings (e.g. Foy et al., 2000), and a recent meta-analysis demonstrated its efficacy, relative to waitlist control, in reducing PTSD symptoms (*d* = 0.56; Sloan, Feinstein, Gallagher, Beck, & Keane, 2013). Indeed, group-based cognitive processing therapy (CPT) yields superior treatment effects for both PTSD and depression symptoms, relative to a present-focused group therapy (Resick et al., 2015) and combined individual and group treatment for adults with childhood sexual trauma (Chard, 2005), with some evidence of a significant effect on complex features (e.g. reductions in dissociation following combined individual and group therapy; Chard, 2005). Other group treatments have also demonstrated promising effects on both core PTSD symptoms (e.g. Sikkema et al., 2007) and the negative affect cluster of symptoms for samples with complex trauma histories (group therapy for incarcerated women; Bradley & Follingstad, 2003; trauma-focussed group therapy; Classen et al., 2011).

However, the majority of group-based interventions have adopted an education and supportive counselling or traditional cognitive-behavioural approach and not explicitly addressed the complex features of CPTSD. This is a vital need within the field, as meta-analysis suggests that current group-based treatments produce smaller effect sizes for indiviudals with more complex trauma histories (e.g. repeated interpersonal trauma; Sloan et al., 2013), compared to mixed trauma samples, suggesting that it may be necessary to explicitly address complex features to maximize therapeutic gains for this group. Dorrepaal et al. (2013) conducted the first study evaluating enhanced PTSD treatment in group format with a specifically CPTSD population: a randomized controlled trial of a 20-week stabilization-focussed cognitive behavioural treatment (CBT) for child-abuse-related CPTSD. The protocol included sessions on psychoeducation, skills training to target the negative affect domain of complex symptoms (learning to tolerate negative emotions and decrease avoidance), and cognitive restructuring. The results demonstrated significant improvements in symptoms of PTSD and CPTSD. We aimed to move beyond this initial study by more explicitly addressing all three symptom domains of CPTSD, with a greater emphasis on memory processing work, and in a shorter-time frame (three rather than five months) that can more easily fit within the time constraints of clinical services.

Here we describe the development and preliminary evaluation of a group intervention for individuals who have experienced repeated interpersonal trauma: an Emotion- and Memory-Processing Group Programme. Developing an efficacious group treatment for PTSD requires careful consideration of the process of intervention, as well as its content (e.g. Foy et al., 2000; Hickling & Blanchard, 1999; Resick & Schnicke, 1993). To implement the phase-based approach, we based our group programme on the Skills Training in Affective and Interpersonal Regulation (STAIR; Cloitre, Cohen, & Koenen, 2006) protocol. STAIR is a phase-based, sequential treatment that was specifically developed to treat women (in individual therapy) who had experienced childhood sexual abuse (Cloitre, Koenen, Cohen, & Han, 2002). The treatment first emphasizes skills training in affective and interpersonal regulation (STAIR) to improve daily life functioning, while the second module (Narrative Story Telling; NST) focuses on the re-appraisal of trauma memories. In NST, patients are asked repeatedly to imagine and then retell the details of their traumatic experiences, which can be difficult to facilitate effectively in a group format due to the risk of trauma narratives triggering responses among fellow group members. Prior research has addressed in a variety of ways, including asking group participants to write their trauma narrative and complete imaginal exposure either while in the group (Beck, Coffey, Foy, Keane, & Blanchard, 2009) or as homework (Castillo et al., 2016). We therefore required participants to complete exposure at home by writing out a narrative of the trauma between sessions, to retain elements of NST from the original protocol. However, we did not ask participants to share a full account of their traumatic experiences within the group sessions.

To facilitate group-based delivery, therefore, we replaced the NST phase of the STAIR programme with a number of different mnemonic control techniques. Given the key role of memory characteristics in predicting prognosis, we aimed to include greater emphasis (relative to STAIR) on memory-processing work, in line with existing evidence-based treatments (e.g. Ehlers & Clark, 2000; Ehlers, Clark, Hackmann, McManus, & Fennell, 2005). Trauma-focused interventions typically involve processing and ‘updating’ trauma memories (e.g. Ehlers & Wild, 2015), and these techniques can be easily implemented in a group format. The second phase of treatment thereby included identifying triggers to traumatic memories and describing the associated meanings, emotions and physiological sensations, cognitive/narrative restructuring, and imagery rescripting. In sum, the final protocol consisted of a skills in affective and interpersonal regulation phase, a memory processing phase, and a skills consolidation phase, delivered over 12 group-based sessions.

We completed a three-group case series of the Emotion- and Memory-Processing Group Programme for complex features of PTSD with female survivors of rape or sexual assault. Guidance on the development of complex interventions (e.g. Medical Research Council [MRC], 2000) recommends that novel clinical techniques are first piloted in small studies, such as case series that serve to establish the promise of a new approach, and are important in refining an intervention (through use of clinician and participant feedback) prior to commencement of trials. The key focus of this study was to develop the novel treatment manual to the point that it may be evaluated in a future feasibility trial, and to provide a preliminary, uncontrolled estimate of any effects of the intervention.

This case series details the delivery of the programme, and provides a preliminary examination of acceptability, feasibility, and potential efficacy of the intervention in reducing symptoms of PTSD, along with measures of complex features, namely emotion dysregulation, dissociation, and interpersonal difficulties. We also looked at changes in posttraumatic cognitions, and depression. Hypotheses for our primary outcomes were: (1) The intervention would show promising acceptability and feasibility, determined by an average attendance of at least eight of the 12 sessions and completion of at least 50% of homework tasks (75% attendance was the rule used within the clinical service from which the participants were recruited, for continuation of psychological treatment. Based on our clinical experience, with this client group, we considered 50% of homework tasks to be the minimum someone could complete and still engage satisfactorily between sessions); (2) Participants would show a reduction in core symptoms of PTSD and associated complex features from pre- to post-treatment. Hypotheses for our secondary outcomes were: (3) Participants would show a reduction in associated symptoms of depression and anxiety from pre- to post-treatment; (4) Participants would show a reduction in scores on process measures of maladaptive cognitions and emotion processing associated with the onset and maintenance of PTSD (Dalgleish, 2004).

## 2. Method

### 2.1. Participants

We conducted three intervention groups in London in 2012–2014. Participants were 15 women aged 19–46 years (*M* = 27.93; *SD* = 6.86). Five women participated in the first group, six in the second group (although one dropped out as she was hospitalized due to suicide risk after the initial assessment, before group began, and her data were set aside) and five in the third group.

Inclusion criteria were that participants experienced complex features of PTSD, had been raped or sexually assaulted in the 12 months prior to the group, and had also experienced at least one prior interpersonal trauma in their lives. Exclusion criteria were insufficient knowledge and understanding of English and current substance dependence. No participants were excluded on this basis.

We operationalized CPTSD by cross-referencing participants’ scores on the Complex Trauma Symptoms Questionnaire (CTSQ; Mendelsohn et al., unpublished). The CTSQ items index the ICD-11 criteria for CPTSD, providing a measure of perceived threat, emotion regulation difficulties, sense of self, self-recognition and agency, interpersonal difficulties, emotional blunting, and meaning attached to the trauma. Responses to each item on the CTSQ ranged from 0 (*not at all*), 1 (*a little bit*), 2 (*moderately*), 3 (*quite a bit*) and 4 (*extremely*). Eleven participants met criteria for at least one symptom on each of the domains (affect, negative self-concept and relational disturbance), determined by a score of two or more on the CTSQ. Three participants met criteria for at least one symptom on two out of three of the domains. One participant described mild complex features, scoring one on a number of criteria on each of the subscales.

Participants were recruited following assessment at The Haven (Sexual Assault Referral Centre) (*n =* 11); by the Sexual Offences Investigative Team (*n =* 1); by the Sexual Health Psychology service (*n =* 2); from the Praed Street Project (supporting women in the sex industry; *n* = 1); from Eaves (a voluntary sector organization supporting female victims of violence; *n =* 1). The group programme was offered as an adjunct to treatment as usual, which involved one or two follow-up medical review and/or support sessions with nurses/support workers at The Haven.

### 2.2. Measures

#### 2.2.1. Symptom and clinical impact measures

PTSD was diagnosed with the Clinician-Administered PTSD Scale (CAPS; Blake et al., 1995). The CAPS is a semi-structured interview which assesses the PTSD diagnostic criteria defined by the Diagnostic and Statistical Manual of Mental Disorders (4^th^ ed; DSM-IV; American Psychiatric Association, 1994 - The CAPS for DSM-IV was used as the CAPS for DSM-V was not available when the first group started.) . The CAPS includes standardized questions to determine frequency and intensity of each symptom in the preceding month. A total severity score for is determined by summing scores for the 17 core symptoms.

The CAPS has good psychometric properties (Weathers, Keane, & Davidson, 2001) and is a sensitive and specific measure of PTSD (Hovens et al., 1994). Inter-rater reliability is high (‘Frequency’ *r* = .92–1.00; ‘Intensity’ *r* = .93–.98; ‘Severity’ *r* = .89; Hovens et al., 1994). Test-retest reliabilities range from .77 to .96 for the three symptom clusters and from .90 to .98 for the 17-item core symptom scale (Blake et.al., 1995). Internal consistency for the severity score was high in the current sample (*α* = .82).

The Complex Trauma Symptoms Questionnaire (CTSQ; Mendelsohn et al., unpublished) is a 49-item assessment measure intended to assess CPTSD symptoms and has been used in previous evaluation of a phase-based approach for treating PTSD in women with a history of interpersonal violence (Cloitre et al., 2014). Internal consistency was high in the current sample (*α* = .97).

Comorbid Axis I diagnoses were determined using the Structured Clinical Interview for DSM-IV disorders (SCID-I; First, Spitzer, Gibbon, & Williams, 2002). The SCID-I assesses DSM-IV diagnostic criteria. The interview takes 45–90 minutes to complete. It is divided into six self-contained modules that can be administered in sequence. The reliability and validity of the SCID-I for DSM-IV is well established and has been reported in several published studies (e.g. Lobbestael, Leurgans, & Arntz, 2011; Zanarini et al., 2000).

The Beck Depression Inventory (BDI-I; Beck, Ward, Mendelson, Mock, & Erbaugh, 1961) indexed symptoms of depression using 21 questions about how the subject has been feeling in the last week. Internal consistency was high in the current sample (*α *= .81). The BDI-I was used for legacy reasons to provide comparability across studies within the research unit.

#### 2.2.2. Process measures

The Post-Traumatic Cognitions Inventory (PTCI; Foa, Ehlers, Clark, Tolin, & Orsillo, 1999) is a 33-item measure of negative and dysfunctional post-trauma cognitions about the self and world. Cognitive models of PTSD emphasize these dimensions as foci of change in cognitive-behavioural interventions (Dalgleish, 2004). The three factors have good test-retest reliability and discriminate well between traumatized individuals with and without PTSD (Foa et al., 1999). Internal consistency was high in the current sample (*α* = .96).

The Difficulties in Emotion Regulation Scale (DERS; Gratz & Roemer, 2004) is a 36-item self-report measure designed to measure emotion dysregulation. Items focus on lack of emotional awareness, lack of emotional clarity, non-acceptance of negative emotions, lack of strategy building, lack of control of impulsive behaviors, and inability to behave in accordance with goals under negative emotions. The DERS has good test-retest reliability, and adequate construct and predictive validity (Gratz & Roemer, 2004). Internal consistency was high in the current sample (*α* = .91).

### 2.3. Description of the intervention

The 12-session group programme comprised: one session involving an introduction to the group and an overview of the subsequent sessions; three sessions focused on emotional awareness and regulation, identifying and labelling feelings, emotion management, distress tolerance and acceptance of feelings, and experiencing positive emotions; two sessions focused on navigating interpersonal problems, exploration and revision of maladaptive schemas, effective assertiveness, awareness of social context (including exploration of other people’s reactions to rape and sexual assault), and flexibility in interpersonal expectations and behaviours; one session for psychoeducation focused on symptoms of PTSD and the impact of trauma on memory; four sessions focused on exposure and mnemonic techniques to better manage trauma memories, identifying triggers to and re-conditioning flashbacks, imagery and nightmare rescripting, narrative restructuring, and the method of loci (Dalgleish et al., 2013; Werner-Seidler & Dalgleish, 2016); and one session for summary and review (see Supplementary materials for an outline of the final 12 session Emotion- and Memory-Processing Group Intervention).

As noted, exposure was not a mandatory part of the group programme. Although we focused on techniques of memory restructuring, such as imagery and nightmare rescripting exercises which involved an element of exposure, we did not facilitate an in-the-moment reliving sessions as a group but, similar to Beck et al. (2009), set an exposure exercise for homework by asking participants to write out a narrative of their traumatic experience(s).

Minor modifications were made following each of the groups in the case series, in line with case series development (MRC, 2000), based on both reflections of the facilitators and specific feedback provided by group members. We offered sessions corresponding to each of the recommended phases for complex presentations of PTSD. Although the initial presentation of the phases was in the linear order originally proposed, development of the manual throughout the case series saw that in Groups 2–3, the phases became more integrated. In particular we continued to use elements of stabilization work in the trauma-processing stage, as group members reported difficulties in practising the regulation of emotions and management of distress before any trauma-focused processing had taken place.

We therefore re-ordered the group sessions to alternate between processing/managing memories and then regulating/coping with the distress, rather than having distinct, linear phases. Facilitators observed ambivalence towards and avoidance of homework tasks and therefore dedicated more time to addressing the reasons for avoidance and included more frequent re-iteration of the importance of between-session exercises. Facilitators also modified the session on ‘interpersonal schemas’ to focus more generally on interpersonal difficulties following a traumatic event as the former was difficult to facilitate in a group within a single session.

The first group was facilitated by a Senior Clinical Psychologist and a Trainee Clinical Psychologist; the second and third groups were facilitated by a Senior Clinical Psychologist and a Mental Health Independent Sexual Violence Advisor. Participants were asked to attend all 12 group sessions, each of which was two hours long, including a 20 minute break. The sessions comprised a combination of clinician-led teaching, group discussions, group exercises, and discussion of homework tasks. Each session began with a review of the homework tasks, an update for any of the group members who had not been present, and then an overview of the current session. Each session ended with a description of the homework tasks for the following week.

### 2.4. Procedure

Ethics approval was obtained from the NHS National Research Ethics Service (reference 11/H0305/1). During pre- and post-intervention assessments, participants completed the study measures individually and face-to-face in a quiet testing room. Following provision of informed consent, participants completed the CAPS and the SCID-I with the assessor, then the self-report questionnaire symptom and process measures. Group sessions took place on a weekly basis in a room in St. Mary’s Hospital, London, UK.

## 3. Results

### 3.1. Description of the sample

The socio-demographic, trauma history, and diagnostic information of the study participants is presented in Table 1 and pre- and post-treatment scores on symptom and process measures are presented in Table 2.

**Table 1.** Sociodemographic, trauma history, and diagnostic information of study participants.

	Group 1	Group 2	Group 3	Total
	(*n* = 5)	(*n* = 5)	(*n* = 5)	(*n* = 15)
Sociodemographic				
Employed (full- or part-time)	3	1	3	7
Full-time Study	0	3	2	5
Education^1^	2/3/0/0	1/1/2/1	0/0/3/2	3/4/5/3
Married/Co-habiting	0	0	0	0
Children	1	1	0	2
Ethnicity^2^	4/1/0/0	1/2/1/1	4/1/0/0	9/4/1/1
Trauma History				
Abuse in Childhood^3^	1/1/1	1/2/1	2/0/2	4/3/4
Abuse in Adulthood^4^	3/5/2	2/5/2	2/5/1	7/15/5
Adulthood Road Traffic Accident	0	0	1	1
Adulthood Natural Disaster	0	1	0	1
Current Axis I Comorbidities				
Major Depressive Disorder	3	3	1	7
Eating Disorder	0	1	0	1
Obsessive Compulsive Disorder	0	0	1	1
Panic Disorder^5^	1	1	2	4

^1^Secondary Education/College/Further Education – Undergraduate/Further Education – Postgraduate

^2^ White/Black/Asian/Mixed

^3^ Sexual/Physical/Emotional

^4^ Domestic Violence/Rape or Sexual Assault/Physical Assault

^5^ Secondary to PTSD Diagnosis

**Table 2.** Pre- and post-treatment scores for symptom and process measures.

	Pre-		Post-			
	*M*	*SD*	*M*	*SD*	*t* (15)	*d*
CAPS Severity	72.92	16.00	56.31	17.28	2.70*	1.18
DERS	116.46	23.42	93.54	16.49	3.97**	1.13
Beck Depression Inventory	26.62	9.06	16.23	4.71	5.82***	1.44
CTSQ Total Score	100.38	43.54	63.92	31.76	4.12**	0.96
Chronic State of Perceived Threat	17.15	6.99	12.38	6.42	2.63	0.71
Emotion Dysregulation	16.85	5.51	12.77	5.72	2.32	0.73
Disturbed Sense of Self	25.92	13.36	16.54	11.58	3.54**	0.75
Lack of Recognition and Agency	9.54	6.05	4.15	3.89	4.46**	1.06
Interpersonal Disturbances	13.15	7.40	10.08	7.27	1.50	0.42
Emotional Blunting	12.38	6.89	6.62	3.64	3.47**	1.05
Lack of Meaning	5.38	3.48	1.38	1.66	4.76***	1.47
PTCI Total Score	171.77	40.83	128.08	29.71	4.41**	1.22
Negative Cognitions about the Self	4.49	1.31	3.28	0.88	3.79**	0.48
Negative Cognitions about the World	5.11	1.29	4.29	1.00	4.37**	0.47
Self-blame	4.15	1.03	2.80	1.19	3.90**	0.52

* p < .05 ** p < .01 *** p < .001

**Table 3.** Reliable change and clinically significant change for combined groups.

			Reliable Change		Clinically Significant Change	
	Test-retest Reliability	*SE* of Change	Criterion	*n* (%)	Criterion	*n* (%)
CAPS – Severity	0.83	9.47	18.55	4 (27)	15 point change	6 (40)
Beck Depression Inventory	0.89	4.25	8.33	9 (60)	18% decrease	10 (75)
Difficulties in Emotion Regulation Scale	0.88	11.47	22.49	7 (47)		
Post-Traumatic Cognitions Inventory	0.82	24.50	48.02	6 (40)		

*n *= number of participants who met the change criterion.

All participants presented with complex features of PTSD, including emotion regulation difficulties, interpersonal problems, impulsive and/or self-destructive behaviour, high levels of dissociation, substance-related problems, and somatic symptoms. Fourteen of the 15 met criteria for DSM-IV PTSD on the CAPS at baseline. The participant who did not meet criteria for PTSD on the CAPS at baseline presented with PTSD symptoms of avoidance and physiological arousal. However, she did not present with reliving symptoms at that time due to very high levels of dissociation and disconnection from her emotions.

All participants had been raped or sexually assaulted in the 12 months prior to the group and had also experienced at least one prior interpersonal trauma in their lives. Participants reported being exposed to between two and *too many to count* past traumatic experiences, as measured by the SCID-I. Seven of the 15 participants had experienced *too many to count* past traumatic experiences due to prolonged abuse in childhood or an adult relationship. Baseline severity on the CAPS was comparable with levels reported in a high dissociation sample of victims of childhood sexual and/or physical abuse (Cloitre et al., 2012), victims of childhood sexual abuse (Chard, 2005), and rape victims with a childhood sexual abuse history (Resick et al., 2003).

### 3.2. Group attendance and homework adherence

The main adherence outcomes of interest were mean number of group sessions completed and percentage of homework tasks completed. There was only one drop out from the intervention (one member of the first group was hospitalized due to suicide risk) and data are presented for the remaining 15 group completers. Participants attended an average of 9.07/12 sessions (*SD *= 2.99; range 2–12). An average of 8.8 sessions were attended in the first group, 8.0 in the second group, and 10.2 in the third group. Across groups, participants completed between five and 28 homework tasks in total (out of 32 tasks set) (*M *= 15.14, *SD *= 8.11). An average of 17.2 homework tasks were completed for the first group, 9.4 for the second group, and 19 for the third group. Overall, eight of the 15 group participants (53%) wrote out a narrative of their traumatic experience in between sessions eight and nine (four in the first group, one in the second group, and three in the third group).

### 3.3. Clinical outcomes

**Figure 1.**
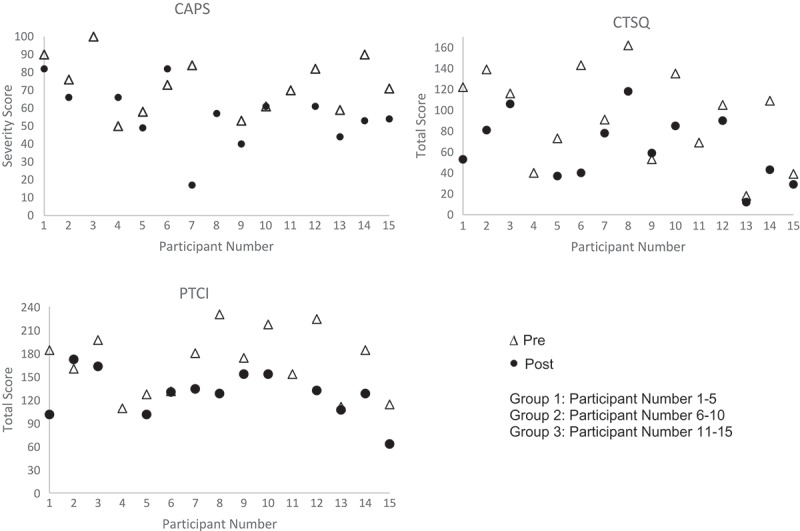
Pre- and post-scores on the CAPS, CTSQ, and PTCI.

The main clinical outcomes of interest were the effect sizes for the symptom and process measures. Prior to the group intervention, 14 participants met DSM-V criteria for PTSD on the CAPS. This reduced to five post-treatment. Table 2 shows the inferential statistics and effect sizes assessing change from pre- to post-treatment on CAPS severity score, the CPTSD measure (CTSQ), BDI, PTCI, and DERS for the three groups combined. Figure 1 presents pre- and post- scores for each participant on the CAPS, CTSQ, and PTCI. Analyses were Bonferroni corrected for multiple testing (*α* = .05/15 = .003).

As can be seen, there were medium to large effect sizes (Cohen, 1977) for improvement on all clinical and process outcomes. Although traditional statistical significance was not the focus of this case series, it is worth noting that these effects reached statistical significance (albeit uncorrected for multiple comparisons) for the CAPS, BDI, PTCI, DERS, and the majority of the subscales of the CPTSD measure.

#### 3.3.1. Calculation of reliable change

Reliable change (Christensen & Mendoza, 1986) indexes whether participants changed sufficiently enough to ensure that the change is unlikely to be due to simple measurement unreliability. The formula for the standard error of change is: SD1√ (2) ×√ (1-rel), where SD1 is the initial standard deviation and rel indicates the test-retest reliability of the measure. The formula for criterion level, based on change that would happen less than 5% of the time by unreliability of measurement alone, is: 1.96 × SD1√ (2) × √ (1-rel). Using this calculation, reliable change was observed for four participants on the CAPS, nine on the BDI, seven on the DERS, and six on the PTCI (see Table 3).

#### 3.3.2. Calculation of clinically significant change

Clinically significant change indexes whether the participant’s score on a given measure has shifted from a score typically associated with the presence of clinical problems to a score typical of the healthy population. On the BDI, clinically significant change was defined as an 18% reduction in total score (Button et al., 2015). On the CAPS, a 15-point change indicates clinically significant change (Weathers et al., 2001). Clinically significant change was observed for six participants on the CAPS and 10 on the BDI (see Table 3).

## 4. Discussion

This case series has demonstrated initial evidence for the feasibility, acceptability, and efficacy of the Emotion- and Memory-Processing Group Intervention. Our primary aim was to determine feasibility and acceptability of the intervention. There was only one drop out from treatment – who was admitted to hospital – and participants attended an average of 9.07 of 12 sessions and completed an average of 15.14 of the 32 homework tasks set. These outcomes provide initial support for the intervention being feasible and broadly acceptable to participants, although a more in-depth qualitative assessment is now indicated.

We also aimed to explore treatment efficacy. Results demonstrated medium to large effect-size improvements on all clinical and process outcomes. Interestingly, effect sizes for change in emotion regulation, a core element of CPTSD, and change in depression symptoms, perhaps as an index of the negative mood component of CPTSD, were in fact larger than overall severity of PTSD symptoms. For the CAPS, BDI, PTCI, DERS, and the majority of the subscales of the CPTSD measure, these reached traditional statistical significance despite the modest sample size. Furthermore, at post-treatment all three groups demonstrated a reduction in the number of participants who met criteria for PTSD, with nine of 14 participants no longer having a PTSD diagnosis post-treatment. Forty percent of participants demonstrated clinically significant change and 27% demonstrated reliable change on the CAPS. A large effect size (*d* = 1.18) for pre-to-post-treatment change in CAPS symptom severity was superior to the moderate effect size reported in meta-analysis of within-group effects of existing group treatments (Standardized mean gain = 0.55) for survivors of repeated sexual violence (as experienced by our sample) (Sloan et al., 2013). Together, these results suggest that the Emotion- and Memory-Processing Group Intervention shows promise for reducing symptoms of PTSD, other complex features of PTSD, and depression in clients with a history of repeated interpersonal trauma.

There are a number of potential strengths of this protocol. The intervention incorporated elements of the phase-based treatment model into a single group programme. We integrated techniques such as imagery- and nightmare-rescripting to help facilitate the processing of trauma memories, along with sessions focused on the consolidation of treatment gains, including ‘emotionally engaged living’, ‘interpersonal emotion regulation’, and the ‘method of loci’ (Dalgleish et al., 2013; Werner-Seidler & Dalgleish, 2016). This study addresses a research gap by examining the effectiveness of a trauma-focused intervention for clients with a history of interpersonal trauma and complex features of PTSD in a group setting, by incorporating the use of mnemonic control techniques and exposure-based interventions. This Emotion- and Memory-Processing Group Programme has promising outcomes as a resource-limited trauma-focused intervention for clients with a history of repeated interpersonal trauma. NICE guidelines currently recommend individual trauma-focused therapy for individuals with PTSD but, as part of a stepped-care approach with limited time and resources available, there is promise for this group intervention.

### 4.1. Limitations and future research

This case series was an important first step in evaluating the clinical utility of the programme, however, there were some limitations to the study. As recommended for early-stage work to explore clinical efficacy (Medical Research Council, 2000), we utilized a small sample size, which limits confidence in the conclusions drawn from the results. Two participants did experience an increase in PTSD symptoms from pre- to post-treatment, however, the small sample size limited evaluation of potential participant characteristics or moderators which may have influenced treatment effects. Finer examination of patient-level change will be an important aspect of future, larger studies. Further, absence of an established diagnostic criteria and psychometric measures for CPTSD limited the availability of rigorous measures with which in index our outcomes. In addition, not all patients met diagnostic criteria for PTSD and although all participants had experienced at least two past interpersonal traumas, only seven participants had experienced prolonged abuse in childhood or an adult relationship. Variation of treatment effects within different trauma-exposed samples thereby warrants further consideration. Other limitations include the lack of follow-up to measure the long-term effects of the intervention and no personality disorder assessments were performed. Moving forward, the increasing emphasis on CPTSD in clinical literature will ensure the availability of sound clinical measures that can be used in future research. As group processes such as peer support, or the normalization of experiences, are likely to contribute to improvement in symptoms, comparison against a control group will be an important next step in developing the intervention. Future studies will need to explore the facilitation of the group programme with a greater number of participants, against control groups.

Further refinement of a treatment protocol is a key aim of a case series, and we identified potential areas in which the intervention may be further developed. Due to concerns identified in the research literature (Beck et al., 2009), direct exposure was not a mandatory part of the group programme. Although we focused on techniques of memory restructuring which involved an element of exposure due to participants being asked to describe their trauma memories (e.g. imagery rescripting), we did not facilitate an in-the-moment reliving session as a group, which would be valuable to consider moving forward.

Avoidance difficulties are a fundamental part of the PTSD presentation and a direct target of trauma-focused interventions. It is difficult to address avoidance in a group setting and to ensure that group participants actually complete homework tasks, such as practicing imagery rescripting or writing out a trauma narrative. Fewer than half of the participants wrote a trauma narrative for homework and, of those who did, it was difficult to determine to what extent they had been *emotionally* engaged with the task at the time. This will thereby need further exploration, as engagement in homework may need to be enhanced to improve treatment effects. Finally, although the group intervention focused specifically on ‘emotion regulation’ and ‘interpersonal emotional regulation’, and achieved good outcomes on a standardized measure of emotion regulation – the DERS – the programme nevertheless only included two-hour sessions focused specifically on each. Group participants had a history of repeated interpersonal trauma and all had some difficulties in emotion regulation and social relationships, and may have benefitted from further intervention in this area.

### 4.2. Conclusion

This study represents an important initial step for building knowledge about effective group-based interventions for individuals who present with complex features of PTSD following a history of interpersonal trauma. Group-based treatments are a practical, cost-effective, and efficacious treatment approach for many psychological disorders, and here we have presented preliminary evidence for a group-based treatment approach, which includes elements (e.g. exposure, memory rescripting) essential to effective treatment for trauma survivors. Evidence from this case series provides a solid platform for future completion of a controlled trial of treatment efficacy, as this protocol presents a novel and promising group-based treatment.
