# Supplementary material for: Developing an Emotion- and Memory-Processing Group Intervention for PTSD with complex features: a group case series with survivors of repeated interpersonal trauma
Source: Eur J Psychotraumatol. 2018 Jul 30;9(1):1495980. doi: 10.1080/20008198.2018.1495980 (PMC6070972; doi:10.1080/20008198.2018.1495980)
Supplement: Supplemental Material [file ZEPT_A_1495980_SM7656.docx]

**Supplementary Materials**

*An outline of the final 12 session Emotion- and Memory-Processing Group Intervention for PTSD with Complex Features*

Session 1: Introduction to the Group

Session 2: Emotional Awareness

Session 3: Psychoeducation - PTSD and Memory

Session 4: ‘Rape’ – Meaning, Myths & Other People’s Reactions

Session 5: Emotion Regulation

Session 6: Emotionally Engaged Living

Session 7: Interpersonal Emotion Regulation

Session 8. Flashbacks – Identifying Triggers and Re-Conditioning

Session 9: Imagery & Nightmare Rescripting

Session 10: Narrative Restructuring & Nightmare Rescripting

Session 11: Method of Loci

Session 12. Summary & Review
